# A Proactive Protection of Smart Power Grids against Cyberattacks on Service Data Transfer Protocols by Computational Intelligence Methods

**DOI:** 10.3390/s22197506

**Published:** 2022-10-03

**Authors:** Igor Kotenko, Igor Saenko, Oleg Lauta, Alexander Kribel

**Affiliations:** 1 Laboratory of Computer Security Problems, St. Petersburg Federal Research Center of the Russian Academy of Sciences (SPC RAS), 39, 14th Liniya, 199178 St. Petersburg, Russia; 2 Department of Integrated Information Security, Admiral Makarov State University of Maritime and Inland Shipping, 5/7 Dvinskaya st., 198035 St. Petersburg, Russia

**Keywords:** time series, fractal analysis, fractal dimension, Hurst exponent, scaling exponent, cyberattacks

## Abstract

The article discusses an approach to the construction and operation of a proactive system for protecting smart power grids against cyberattacks on service data transfer protocols. It is based on a combination of computational intelligence methods: identifying anomalies in network traffic by evaluating its self-similarity, detecting and classifying cyberattacks in anomalies, and taking effective protection measures using Long Short-Term Memory (LSTM) and Gated Recurrent Unit (GRU) cells. Fractal analysis, mathematical statistics, and neural networks with long short-term memory are used as tools in the development of this protection system. The issues of software implementation of the proposed system and the formation of a data set containing network packets of a smart grid system are considered. The experimental results obtained using the generated data set demonstrated and confirmed the high efficiency of the proposed proactive smart grid protection system in detecting cyberattacks in real or near real-time, as well as in predicting the impact of cyberattacks and developing efficient measures to counter them.

## 1. Introduction

World trends in information and telecommunication technologies based on digital methods of information transmission, processing, storage, presentation, and protection consist in the mutual penetration and “merging” of information and telecommunication systems not only at the level of technologies for their development and operation, but also their structural and functional association. In this case, the term “data transmission network” (DTN) is widely used [1].

There is an integration and convergence of networks and services. This provides users with access to any service available in multiple networks, due to the flexible possibilities for their processing and management. As a result, on the one hand, the efficiency, reliability, economic benefits, and sustainability of the DTN operation increase. On the other hand, it gives the malefactors the opportunity to act by implementing cyberattacks (CAs) [2].

There are many reasons why it becomes possible to implement CAs. It can be an operating system or other software that has not been updated in time. In addition, outdated security features or vulnerabilities inherent in poorly protected network protocols can lead to attacks. As a result, an attacker can perform various malicious actions, such as blocking network communication, making unauthorized access to DTN devices, controlling traffic, changing network device parameters, and other actions.

The category of dangerous services includes services whose placement on the perimeter carries increased risks: file system access services, Remote Procedure Call (RPC), directory services, printers, virtualization system service interfaces, Virtual Private Network (VPN), DTN-specific systems, network device services, Telnet, Secure Shell Protocol (SSH), Remote Desktop Protocol (RDP), Virtual Network Computing (VNC), and others [3]. In addition, it should be noted that security flaws in service protocols that lead to traffic redirection and interception of network configuration information, security flaws in the NetBIOS Name Service (NBNS) and Link-Local Multicast Name Resolution (LLMNR) protocols, as well as the use of open (unsecured) data transfer protocols in modern DTNs, have a high level of risk [4]. As practice shows, the vast majority of successful CAs are based on the exploitation of vulnerabilities in some resources that should not be available on the network perimeter [5].

This fully applies to information systems in the energy sector, built according to the Smart Grid (SG) concept. In accordance with this concept, the priority areas for the development of DTN in the energy sector for the coming years include [6]:

- widespread introduction at new and upgraded measurement points of intelligent measuring instruments—“smart” meters with the function of remote control of the load profile of the measured line and measuring transducers with standard communication interfaces and protocols that comply with information security standards;
- installation at each large facility connected to the power grid, advanced automated information-measuring systems operating in real-time;
- creation of a wide network of integrated communications based on various communication lines;
- implementation of automated production management systems in energy companies.

The application of modern information technologies (ITs) makes it possible to significantly increase SGs operation efficiency, making them more reliable and economical, which, in its turn, leads to a reduction in the cost of power reproduced or distributed by them. However, at the same time, there are opportunities to influence SGs by various CAs. A consequence of this impact is the appearance of anomalies in the SG network traffic [6].

Detecting CAs in SGs is quite a complex task. It is necessary to constantly monitor security and control network traffic in order to detect anomalous activity in it. If traffic anomalies are detected, it is necessary to analyze a large number of routes in the network, where sharp fluctuations in traffic, delays in its transmission, or large packet losses appear. At the same time, a high quality of telecommunications service and application service should be ensured. All of this is the motivation for finding and developing new methods and approaches for CAs detection in SGs. Such approaches in this article include an approach that combines several methods of computational intelligence: the use of fractal analysis, statistical methods, and machine learning.

It should be noted that a fairly large number of classification and prediction methods mostly related to anomaly detection [7,8] are currently known and widely used. In particular, regression-based methods have performed well. These include non-parametric regression and classification tree method (CART) [9], multivariate adaptive regression splines (MARS) [10,11], support vector regression (SVR) [12] and others. Regression-based methods demonstrate high classification and prediction performance if their parameters are well-tuned. In some cases (for example, for MARS and SVR), it is proposed to use genetic algorithms to adjust the regression parameters.

However, this does not allow one to speak about the possibility of early detection of CAs. Therefore, it is believed that the most effective method of classification and prediction is the Long Short-Term Memory (LSTM) neural network algorithm. The LSTM property of recurrence allows an Artificial Neural Network (ANN) to “refer” to the results of its work in the past, to analyze predictions. Thus, the content of decisions made to protect SGs from CAs will depend not only on the results of initial training of the LSTM network, but also on the results of further operation of this network in the flow [13,14].

The key parameter of fractal analysis is the Hurst exponent. This measure is used in the analysis of time series. The Hurst exponent shows the amount of delay in the time series between two identical pairs of values. The bigger it is, the smaller this parameter is. To find this parameter, it is first necessary to check the process under study for stationarity. The presence or absence of stationarity of the process influences the choice of the algorithm by which the scaling index can be calculated.

Fractal properties are more pronounced in non-stationary network traffic, which is predominant in SGs on large data scales. On small amounts of data, or in application layer protocols of the TCP/IP (Transmission Control Protocol/Internet Protocol) model, network traffic can be stationary and show less fractal properties. In this case, machine learning methods are used for further analysis.

Thus, in order to detect and classify the CAs, first, it is necessary to determine whether the traffic is stationary or non-stationary. Next, you should calculate the Hurst exponent (i.e., determine the presence of the self-similarity property in the traffic). In the final stage, anomalies are detected and measures are developed to protect the SG using LSTM [6,15].

The main contribution of this work is as follows: (1) the structures of long-term dependencies in the SG traffic were studied, which made it possible to identify its characteristic features in the interest of the early detection of CAs; (2) a new approach to the detection of CAs based on the study of the fractal properties of traffic has been proposed; (3) the LSTM structure was substantiated, which makes it possible to detect SC with a probability of 0.99; (4) a software prototype was developed that implements the proposed system, and a dataset was generated with SG traffic containing anomalies from the impact of both known and unknown CAs; (5) a comparison was made with other methods of machine learning in identifying the fact of the impact of CAs; (6) an experimental evaluation of the proposed system was carried out, showing its rather high efficiency.

The significance of the new contribution lies in the fact that the detection of CAs is performed using an autoencoder trained on the basis of the reference data of the SG operation and the information exchange in it, taking into account all deviations from the SG regular operation. During operation, the autoencoder is additionally trained by a validated neural network. The result is a generative adversarial network in which neural networks learn from each other. This made it possible to reduce the time for detecting anomalies in network traffic and increase the probability of detecting unknown computer attacks up to 0.8.

The proposed approach has a number of methodological and technical limitations. Methodologically, the approach is limited to the use of the most well-known methods of fractal analysis, which include the Dickey–Fuller test, the rescaled range (R/S) analysis, and the Detrended Fluctuation Analysis (DFA) method, and one of the most promising ANN models, which is the LSTM model. The technical limitations are determined by the computing power of the environment, on which the autoencoder and the neural network are trained, as well as by obtaining a reference sample of the SG operation, taking into account all deviations from the normal operation mode. The training quality of the generative-adversarial network and the detection efficiency of known and unknown CAs depend on the quality of the sample made. Since the autoencoder is additionally trained by the ANN, an incorrect sample can break the ANN operation logic.

The novelty of the results obtained lies in the fact that, based on experimental studies, the best method for determining self-similarity for non-stationary and stationary time series is substantiated, which allows detecting changes in traffic with high accuracy and quickly, and the structure of the LSTM neural network is determined, which provides high accuracy and can sufficiently quickly predict the impact of CAs and allows developing proactive protection measures. This is a significant advantage of the proposed system.

This work is a continuation of the studies published in [6] and is devoted to testing the possibility of using fractal analysis methods for detecting CAs against smart power grids. The difference in this work lies in the addition of neural network analysis methods using LSTM networks to fractal analysis methods. This approach, in contrast to [6], allows one not only to detect anomalies in the SG traffic, but also to identify the types of CAs that are the causes of these anomalies.

For this purpose, we propose the structure of an autoencoder trained on the normal data of the SG network operation, considering possible deviations from the SG normal operation. The complex use of fractal analysis methods, autoencoder, and LSTM network forms the basis of the SG proactive protection system, which is able to detect both known and unknown CAs.

The article has the following further structure. Section 2 is devoted to the analysis of known works in the research field. The theoretical foundations of the proposed proactive CA detection system, which is based on the fractal analysis of the network traffic and their subsequent processing using LSTM networks, are discussed in Section 3. A general description of the proposed system is given in Section 4. Section 5 presents the experimental evaluation results. Section 6 is a discussion of the experimental results and their comparative evaluation. Final conclusions and directions for further research are contained in Section 7.

## 2. Related Work

Fractal analysis, which studies the properties of self-similarity, is currently in a phase of active development. Fractal analysis is widely used for state monitoring problems, in which time series are investigated. For example, [16] proposes to use the R/S analysis method to analyze the self-similarity of time series. The self-similarity properties of the Voice Over Internet Protocol (VoIP) traffic are modeled and studied in [17]. The fractal dimension, which is an additional measure with respect to the Hurst exponent, is investigated in [18]. The reasons explaining the presence of self-similarity properties in telecommunication traffic are given in [19]. However, the main area of research in all these papers, as a rule, is both VoIP-telephony and economic systems.

At the same time, it should be noted that there are few practical experiments aimed at studying the fractal properties of the network traffic in information and telecommunication systems. Among such works, we can single out works [20,21,22]. However, [20] considers the mobile communication traffic generated by cellular stations. The authors conclude that the properties of self-similarity are inherent not only in computer and telecommunications networks, but also in the radio waves on which cellular stations operate. Self-similarity of motion is considered in [21,22]. To detect it, it is proposed to use visual cues, which allow one to find similar areas on the motion graph. These areas allow one to identify self-similar processes.

One of the first works, in which the main attention was paid to the self-similarity property of the network traffic, is the work [11]. It significantly changed the existing ideas about the processes taking place in information and telecommunication networks. These issues will be discussed in more detail in the next section. In addition, we should mention some works in which the mathematical models designed to describe self-similarity in network traffic have been proposed and investigated [23,24]. However, these works cannot be considered exhaustive, since they did not consider the issues of CA detection. Consequently, we can assume that our work, on the one hand, further develops the theoretical positions achieved in the study of the fractal properties of the network traffic. On the other hand, it develops the well-known solutions further in the direction of creating a method that makes it possible to detect network traffic anomalies caused by the impact of CAs.

At the same time, it should be noted that when considering threats to SG security, one should be guided by the following two indicators that characterize these threats. The first indicator is the probability of the threat realization. The second indicator is the potential damage that can be incurred by the power company in case of security threat realization [6,14]. Considering and combining these indicators, it is possible to substantiate the choice of the most acceptable threat models for SGs and to create protection systems for them, in which the decisions made would allow one to minimize security risks.

The first group [25,26,27,28,29,30,31] summarizes the techniques based on quantitative criteria. Thus, [25] proposes to use the acceptable level of the possible damage from information and technical impact on SG resources and the assessment of the profit factor from investments in protective measures as a measure to rank threat models. Quantitative methods comply with the requirements of ISO 27,001 and 27,002, NIST, and COBIT IV [26,27]. Although these methods take into account the predetermined risk appetite, they do not consider the variability in the construction of the SG protection system [28]. In addition, one of the significant disadvantages of the aforementioned methods is the high cost and complexity of their implementation [29]. At the same time, the complexity of quantitative methods is due to the need to take into account each potential security threat in the formation of options for counteracting CAs and developing solutions to eliminate the consequences of CAs [30]. For these purposes, [31] proposes to perform the ranking of security SG risks. Although this technique is undoubtedly of interest, it contains a number of negative factors associated with the problem of cloud resources.

The second group of methods [32,33,34,35] received the generally accepted name of qualitative methods. These methods apply qualitative indicators and criteria for the characterization of SG security threats. The essence of qualitative methods is the search for such a solution, in which the necessary balance is observed between the costs spent on building the protection system and the effect achieved with its help. Such methods form a direction called Cost/Benefit Analysis. In these methods, basically, different positions of the game theory, for example, matrix games are used. Speaking about the disadvantages of qualitative methods, it is necessary to point out their comparatively high computational complexity. It is due to the need to conduct a security risk analysis in order to make an economic justification for the introduction of protection mechanisms and means for various threat models into SG protection systems. Methods using qualitative criteria are similar in essence to the Facilitated Risk Analysis Process (FRAP) method [36,37].

The third approach [38,39,40,41] is an integrated one; it rationally combines the first and second groups of methods. Most often, the methods of this group find their application in small and medium-sized energy companies. The disadvantages of these methods include, as a rule, a very small amount of analytical data characterizing the potential damage under the given models of CA realization, as well as insufficiently complete risk assessment.

Besides, the works [42,43] present a structured approach to assessing the threat model for information and telecommunication resources (methods “CRAMM”, “MEHARI”). Here an integrated representation of the information security threat parameters is performed, but the specificity of building the SG protection system is practically not considered.

There is a well-known methodology for managing the information security system—Microsoft Security Assessment Tool (MSAT) [44,45]. This tool uses a mechanism for ranking threat models. In addition, the tool provides countermeasures for SG security threats and evaluates their effectiveness. However, the tool is not scalable enough. That is why in SG it is usually implemented in local computing networks or in companies with fewer than 1000 employees. The Risk Management Guide [34] is the basis for this tool’s design and operation. Among the main functions performed by the tool, in addition to risk assessment and decision support, one can include performance monitoring and evaluation [13].

Thus, all the considered approaches to CA early detection and prediction are based either on an in-depth analysis of possible risks (probable damage), or on a selective ranking of threats and defenses. In our opinion, these approaches are insufficient to protect SGs from CAs. For this reason, this article discusses the key points of building an improved system for CA early detection, which can be called proactive. The proactivity of the system lies in the fact that it implements anomaly detection in the network traffic, their identification, and classification based on fractal analysis methods, and a neural network with a long short-term memory, which allows one to reduce risks in the implementation of CAs. The consideration of the proposed system is architecture-oriented. On the one hand, it goes beyond an abstract representation, and on the other hand, it does not pay much attention to technical details. We conclude this article with a detailed look at the proposed active security solutions for SGs and their implementation.

## 3. Theoretical Foundations of the Proposed System

### 3.1. Stationarity of Temporary Traffic

Consider the autoregressive process in general terms:

(1) $$x_{t}=C+\sum_{i=1}^{p}\varphi_{i}X_{t-i}+\in_{t}+\sum_{i=0}^{q}\theta_{i}\in_{t-1}$$

where $\varphi_{p},\theta_{q}\neq 0$ are the model parameters, C is a constant, $\in_{t}$ is a white noise, $x_{t-i}$ is a previous element of the time series.

The model can be interpreted as follows: the current value depends on past values up to lag *p* and on current and past external shocks up to lag *q*. To write the autoregressive process, it is convenient to use the lag operator *L*. The lag operator allows one to obtain the values of the elements of the time series based on several previous values. A lag operator of order *i* is an operator that shifts the value of the time series $x_{t}$ by *i* values back, i.e., $L^{i}:x_{t}\to x_{t-i}$. Using lag operators, the autoregressive process can now be written more visually as follows:

(2) $$x_{t}=C+\sum_{i=1}^{p}\varphi_{i}L^{i}X_{t}+\in_{t}+\sum_{i=0}^{q}\theta_{i}L^{i}\in_{t}$$

Let us rewrite as follows, moving the autoregressive part to the left side of the equality:

(3) $$\left(1-\sum_{i=1}^{p}\varphi_{i}L^{i}\right)x_{t}=C+\left(1+\sum_{i=1}^{q}\theta_{i}L^{i}\right)\in_{t}$$

Now we introduce two polynomials of degree *p* and *q*:

(4) $$\varphi \left(z\right)=1-\sum_{j=1}^{p}\varphi_{j}z^{j}=1-\varphi_{1}z-\varphi_{2}z^{2}-\ldots -\varphi_{p}z^{p}$$

(5) $$\theta \left(z\right)=1+\sum_{j=1}^{q}\theta_{j}z^{j}=1+\theta_{1}z+\theta_{2}z^{2}+\ldots +\theta_{p}z^{p}$$

where $\varphi_{j}$ and $\theta_{j}$ are polynomial coefficients depending on the monomial z, which is a complex number.

Then the autoregressive model can be formally written as $\varphi \left(L\right)x_{t}=C+\theta \left(L\right)\in_{t}$, where φ(L)x is the autoregressive part of the polynomial, and $\theta \left(L\right)\in_{t}$ is the moving average part.

The time series is stationary if all roots of the autoregressive polynomial $\varphi \left(z\right)=1-\varphi_{1}z-\ldots -\varphi_{p}z^{p}$ lie outside the unit circle of the complex plane $\left|z_{j}\right|>1$ (that is, they are greater than 1 in absolute value). The inequality $\left|z_{j}\right|>1$ is satisfied if $\left|\varphi_{j}\right|<1$. Consequently, the relation $\left|\varphi_{j}\right|<1$ is a condition of stationarity of the autoregressive process.

In addition, for a stationary process, the average is constant in time $Ex_{t}\equiv \mathrm{const}$, i.e., the time series does not have a trend, and the covariance between different elements of the time series depends only on how far they are from each other in time. In other words, the covariance depends only on the lag *h* $\mathrm{cov}(x_{t},x_{t+h})=\gamma \left(h\right)$. The value *h*, which characterizes the difference in time between the elements of the time series, is called a lag variable or delay. Since $\gamma \left(0\right)=\mathrm{cov}(x_{t},x_{t})=\mathrm{Va}r\left(x_{t}\right)$, the variance of the stationary time series also does not change with time.

Thus, to test the hypothesis of stationarity of the series, the generalized Dickey–Fuller test is used, and to determine anomalous activity in the network, we are guided by the principle of self-similarity for non-stationary traffic, which is violated when anomalous activity occurs. R/S or DFA algorithms are used to calculate the self-similarity property. The first one is faster, and the second one is more accurate. The process is non-stationary if these conditions are violated.

### 3.2. Self-Similarity Analysis in Network Traffic

Many natural processes are characterized by distributions with heavy tails. Such distributions include the Pareto, Cauchy, Levy, and Weibull distributions, as well as the lognormal distribution. An important feature of exponential distributions is the realization of events that deviate strongly from the norm. Such distributions can be applied to model network traffic intensities and rates that have large, theoretically infinite variances.

The lognormal distribution is the earliest model of self-similar traffic. It is used to model network packet arrival intervals and file sizes transmitted [46]. The Weibull distribution is applied to model the arrival processes of FTP protocol blocks. The Pareto distribution is used to model the intervals between requests to web resources and VoIP traffic [17,46].

In our work, network traffic is considered as an aggregation of several flows from different sources. The aggregated flow, jointly transmitted over communication channels with infinite variance, leads to self-similar network traffic, which is described by the model of fractal Brownian motion [47]. If one of the partial flows has self-similarity when aggregating flows, then the resulting aggregate flow will also have self-similarity [24]. In this case, self-similarity is preserved when aggregating flows coming from both homogeneous and heterogeneous traffic sources.

Fractal Brownian motion is easily applicable to modeling self-similar traffic. The process *X*(*t*) is called a fractal Brownian motion with the parameter *H*, 0≤H≤1, if the increments of the random process have a Gaussian distribution:

(6) $$P\left(\Delta X<x\right)=\frac{1}{\sqrt{2\pi }\delta_{0}\tau^{H}}\int_{-\infty }^{x}\exp\left[-\frac{z^{2}}{2\delta_{0}^{2}\tau^{2H}}\right]dz$$

where $\delta_{0}$ is a diffusion coefficient.

Wherein: 

(1) X(0)=0;

(2)$\;\Delta X=X\left(t_{2}\right)-X\left(t_{1}\right)$ has a normal distribution with zero mean and variance—$\delta^{2}{\left(t_{2}-t_{1}\right)}^{2H}$, 0 ≤ *H* ≤ 1, where *H* = 0.5 indicates a random row. The events are random and uncorrelated. The range of accumulated deviations should increase in proportion to the square root of time.

As noted above, the Hurst exponent *H* is a measure of the self-similarity of the process. If the process has strongly pronounced fractal properties, then *H* approaches unity. In the absence of self-similarity *H* = 0.5 [15]. In this case we speak about fractal Brownian motion, which coincides with the classical Brownian motion and imposes a large noise on the time series [16,26,27].

### 3.3. Detecting Anomalous Bursts Using Machine Learning Techniques

**Figure 1:**
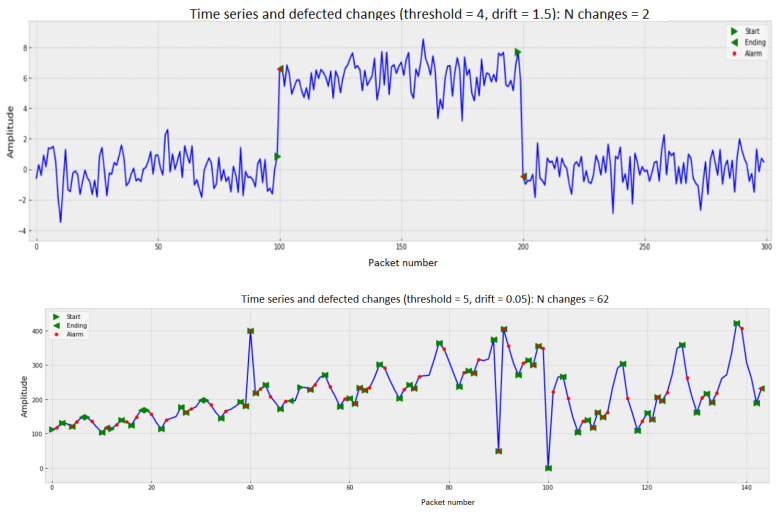
Cumulative sums.

**Figure 2:**
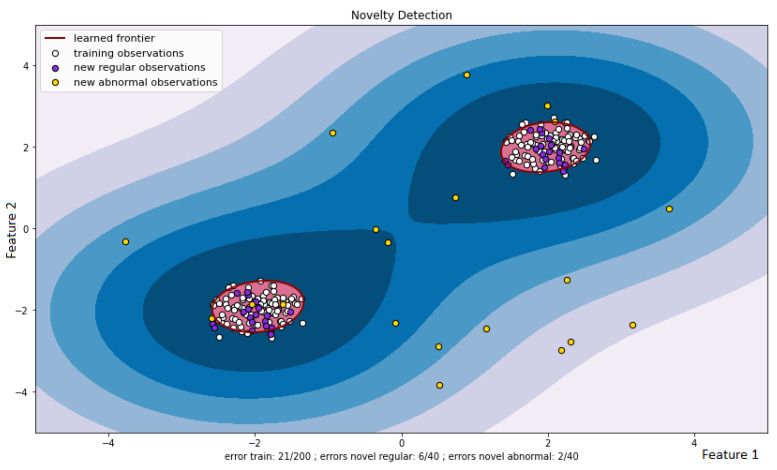
Support Vector Machine.

**Figure 3:**
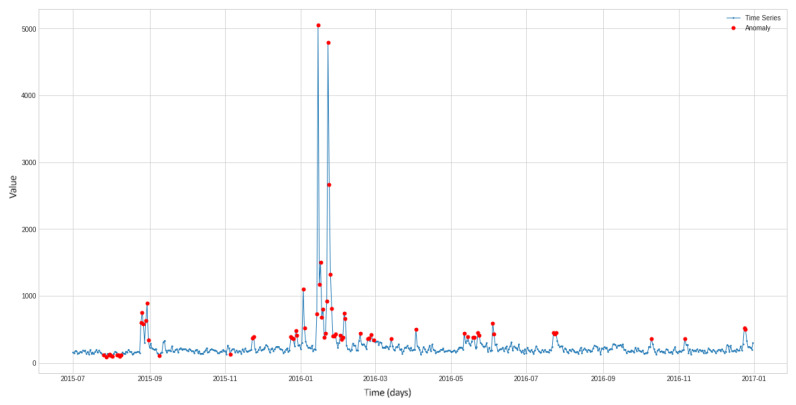
Isolated forest.

There are many ways to identify anomalies. Figure 1, Figure 2 and Figure 3 demonstrate the operation of the most popular machine learning algorithms tested on time series generated using an autoregressive integrated moving average model.

Figure 1 shows different variants of time series with different values of the threshold and drift parameters, on which anomalies (changes) are detected using the cumulative sum method. Detected changes are marked with red dots. It can be seen that the number of detected anomalies in the time series can be different (from two to 62).

Figure 2 outlines the results of anomaly detection using the support vector machine. Each observation has two normalized coordinates—Feature 1 and Feature 2. Feature 1 plays the role of the “Packet Delay” characteristic, and Feature 2 displays the “Bandwidth” characteristic. The white dots indicate the observations that were used in the training set. Their boundaries are marked with red lines. Testing a new dataset using a trained Support Vector Machine (SVM) classifier results in a division of observations into normal (purple dots) and anomalous (yellow dots).

Figure 3 depicts the results of anomaly detection in a time series using an isolated forest. At points where there are anomalies (red dots), the time series changes the parameters of its distribution. An isolated forest is good at detecting these changes.

As can be seen from the figures, the algorithms do an excellent job of detecting anomalous outliers. In this case, the anomaly manifests itself in the form of the non-stationarity of some observed time series. These are not only instantaneous jumps in the measurement amplitude, but also slow trends that are practically invisible during the observation period.

**Figure 4:**
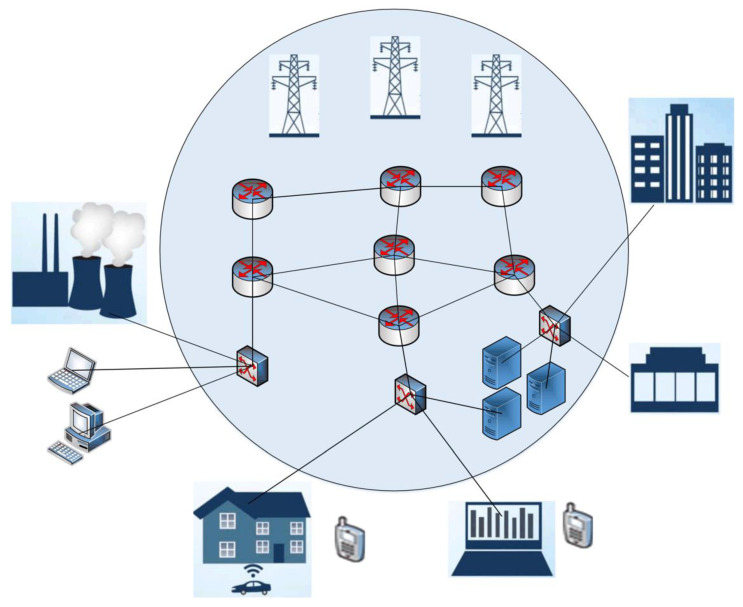
Cyber polygon designed to collect network traffic and analyze its security.

However, when testing the above algorithms on real network traffic, it turned out that outliers are not always anomalous. Therefore, to study the features of anomaly detection in SG traffic, a cyber polygon was developed, shown in Figure 4.

About 30 types of CAs were carried out in the cyber polygon and 40 GB of legitimate traffic was generated. Network traffic was redirected to Security Onion and written to pcap files. From this traffic, a dataset was formed using Netsniff-ng and Bro. The attacks were carried out using the Kali Linux distribution against known vulnerable services deployed in the central part of the scheme. Next, a search for anomalous bursts was carried out using the algorithms of cumulative sums, isolated forest, and SVM. Despite the fact that these algorithms do an excellent job of finding anomalous bursts, it was found that bursts are not always anomalies.

For data packet transmission, modern standards, protocols, and technologies for high-speed electrical networking were considered, such as:

- Fast Ethernet and Gigabit Ethernet suite of standards, which define wired connections and electrical signals at the physical layer, and packet format and medium access control protocols at the data link layer;
- wireless transmission standards based on GSM/EDGE and UMTS/HSPA, which allow data rates of 100 Mbps (with mobile subscribers) and 1 Gbps (with fixed subscribers);
- IEEE 802.11 local wireless networks, which use infrared radiation and radio waves as the physical transmission medium.

The scenario according to which the message packets are transmitted is stationary in this case. Sensors were installed in homes, shops, and offices. The Leningrad Nuclear Power Plant (LNPP) and the South-Western Thermal Power Plant (SWTPP) acted as sources of electricity. The control over the security of the SG network was ensured by the operator (the incident monitoring system).

The intercepted traffic was a data set containing information processed by the operators and dispatching systems of the SG power system. This information included the following parameters:

- equipment state parameters;
- load parameters for transformers;
- parameters of the distributed measurement system;
- power quality parameters;
- information about the locations of damage and denial of service;
- power factor values;
- profiles and forecasts of electricity consumption, as well as some other parameters.

The SG telecommunications network was considered one of the types of computer networks. Therefore, we assumed that the telecommunications SG network has the self-similarity property. Our assumption was later confirmed in the course of experiments.

Based on the fact that the greatest amount of information is stored and transmitted by the operators and dispatchers of the SG power system, the monitoring system, as well as the LNPP and SWTPP data transmission networks with control system were selected as the object for the implementation of the CA.

It was assumed that the ports in the edge network equipment have a bandwidth of 1 Gbit/s [6] and operate over the Ethernet protocol. Traffic generation was performed using the developed simulation model. The GNS3 framework (Galaxy Technologies, LLC., https://www.gns3.com/ (accessed on 20 September 2022)) was used to build this model.

Table 1 shows a list of the main attributes that were included in the dataset generated with GNS3.

The total number of different Flow.ID values in the dataset was 1,522,917. Address 10.200.7.217, corresponding to SWTPP, was used as Source.IP for 7% of all entries. The value 10.200.7.218 corresponding to LNPP was in the Source.IP parameter for 8% of all records. The rest 85% of the entries had other Source.IP values. The generated addresses 10.200.7.7 and 10.200.7.8 were used as Destination.ID field values in 9% of all records. They corresponded to computer networks located in the “Passage” and “Gostiny Dvor” shopping centers. The remaining 82% of the records had other values of the Destination.IP field (their number was 2,939,141).

The self-similarity analysis was performed on the time series formed from the values of the Packet.Length.Mean field. This attribute in the generated dataset had 10,700 unique values. The most frequent values were 267.5 and 243.5 [6].

Two CA types impacted the SG’s simulated infrastructure. These attacks were a DDoS attack and a “Network and Vulnerability Scanning” attack. Traffic impacted by the first type of attack was simulated using the IXIA’s IP network test equipment. A distributed network and SYN Flood, Ping Flood, and UDP Flood methods were used to implement the first CA type. The second CA type was simulated using IP network scanning tools Nmap and Xspider. The probing method was used to implement this attack. According to this method, Nmap or Xspider network scanner simulates an attack aimed at active exploitation of the analyzed vulnerability.

A SYN Flood attack was simulated as follows. The attacker (client) used the standard way of opening TCP connections. For this purpose, a SYN packet was sent to an open server port. After receiving and processing this packet, the server returned the SYN-ACK packet. The SYN-ACK packet contained client-specific data taken from the Traffic Control Unit (TCU) store. In normal circumstances, the client sends back an ACK packet, which serves as confirmation and allows a TCP connection to be opened. However, in the case of a SYN attack, the attacker generated and sent multiple repeat requests to the server with spoofed IP addresses. The server, being the target of the attack, treated them as legitimate requests. It processed them all and tried to open a TCP connection for them. Ping Flood and UDP Flood attacks were implemented in a similar way.

Thus, under conditions of CAs, the data set included the additional attributes (flags) shown in Table 2.

The type of attacks being modeled was considered during dataset generation and determined the FIN, SIN, RST, and ACK flags values. For example, the number of single SIN and ACK flag values increased if the “Network and Vulnerability Scanning” attack was simulated. Thus, in the traffic used for the experiments described in this article, single SIN flag values accounted for 20% and single ASK flag values for 60% of all values [6].

**Figure 5:**
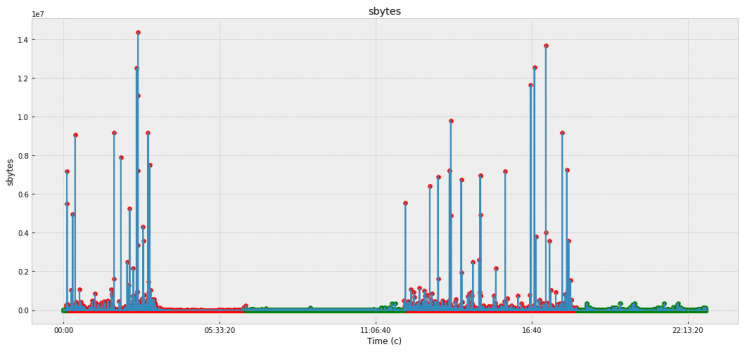
Retransmitted or dropped packets.

**Figure 6:**
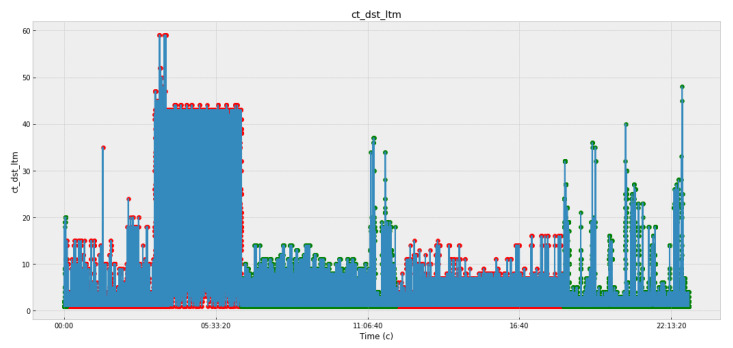
Number of connections to the server.

**Figure 7:**
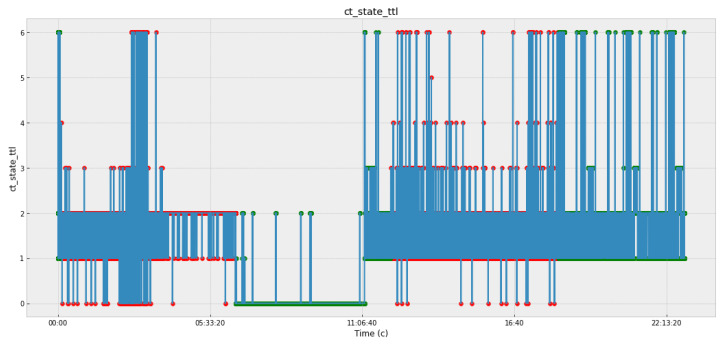
States of TCP header parameters during the lifetime of an IP packet.

**Figure 8:**
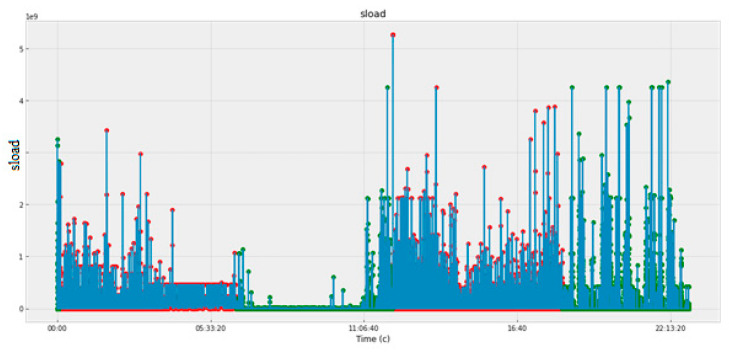
Packet transfer rate, bit/sec.

Figure 5, Figure 6, Figure 7 and Figure 8 and Table 3 present the data obtained at the cyber polygon on the protocols and network parameters under study. Table 3 shows the network protocol parameters that were studied. Figure 5 shows statistics on retransmitted or dropped packets. Figure 6 demonstrates the dynamics of changes in the number of connections to the server. Figure 7 depicts how the state of the TCP header parameters changed over time during the lifetime of the IP packet. Figure 7 shows how the packet rate has changed over time.

In Figure 5, Figure 6, Figure 7 and Figure 8, anomalous packets are marked with red dots and normal (legitimate) packets are marked with green dots. As can be seen from these figures, many bursts are legitimate and, conversely, in many places where there are no bursts, there are anomalies. Therefore, the issue of timely detection of bursts of traffic in SG, identification of anomalous ones from them, as well as classification of detected anomalies in order to predict the fact of the impact of CAs and develop effective countermeasures is an acute issue.

### 3.4. Anomaly Detection with Classifiers

**Figure 9:**
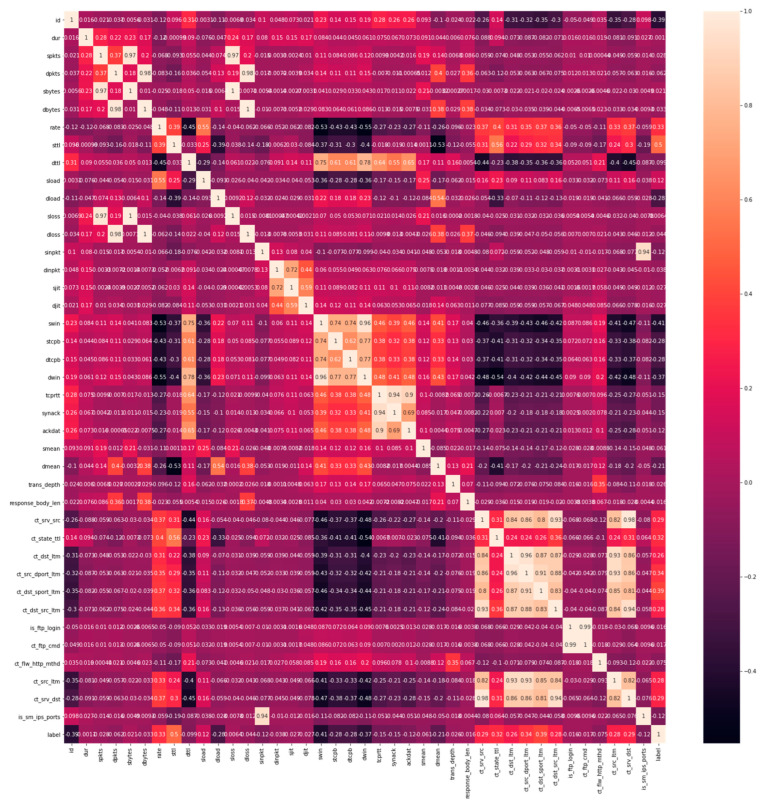
Correlation matrix.

To evaluate the effectiveness of popular classifiers, a dataset was formed containing correlated parameters with anomalous queries. For this purpose, a correlation matrix was built (Figure 9).

**Figure 10:**
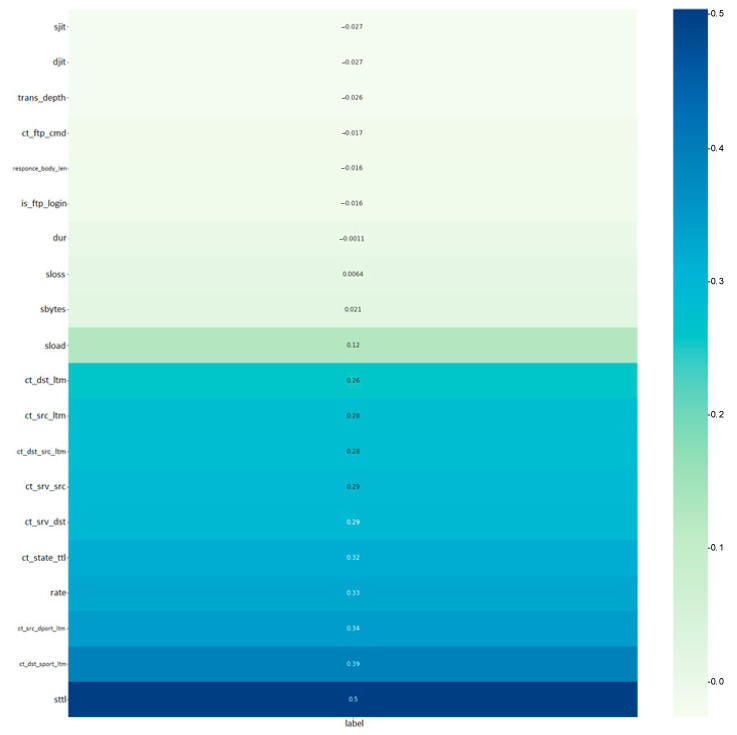
Most correlated parameters.

The label parameter (the last parameter in the correlation matrix) is an indicator showing the presence of anomalies. From the parameters presented in the correlation matrix, 20 parameters were selected that are most correlated with anomalies. They are outlined in Figure 10.

**Figure 11:**
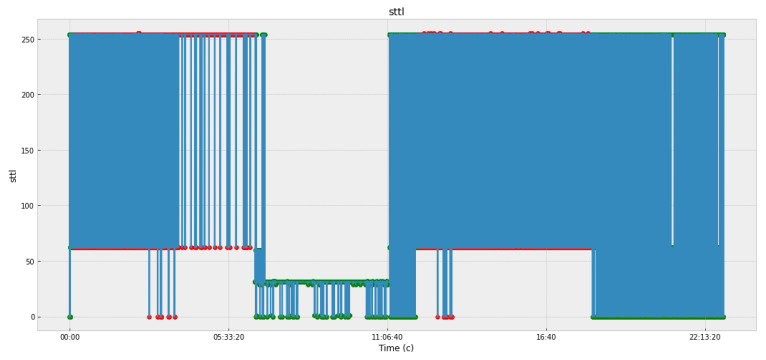
Packet lifetime from source to sender.

The sttl parameter, which indicates the lifetime of the packet during its transmission from source to sender, is most affected. The dynamics of this parameter with indications of anomalies (shown in red dots) are demonstrated in Figure 11. The figure shows the dynamics of the value, which tells the local server how long to keep the packet information in the IP protocol.

**Figure 12:**
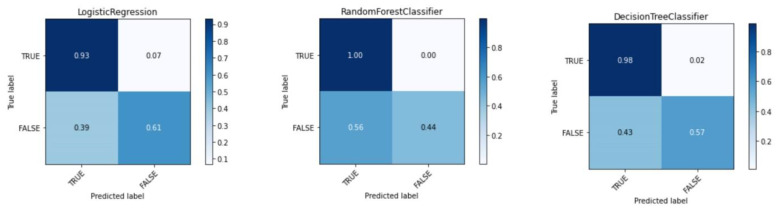
Comparing the efficiency of classifiers.

Logistic regression, random forest, and decision tree were chosen as classifiers that were used at the cyber polygon. These classifiers are not chosen by chance. They have been widely used in the works of many researchers and in many cases provide a sufficiently high classification efficiency, including in ensembles of classifiers. To evaluate their effectiveness, a confusion matrix was calculated. It was used to determine not only the accuracy, but also the number of false positives. The results of the selected classifiers are shown in Figure 12. It can be seen that despite the high efficiency of the classifiers used at the cyber polygon, they all had a large number of false positives.

For Logistic Regression the First Kind Error is 39%. The Random Forest algorithm has 56% of false positives. The Decision Tree algorithm also shows quite aggressive behavior, which is caused by the First Kind Error, equal to 43%.

The obtained results confirm that the main problem of known classifiers is the poor ability to recognize previously unknown anomalies.

## 4. General Description of the Proposed System

### 4.1. Stages of System Operation

**Figure 13:**
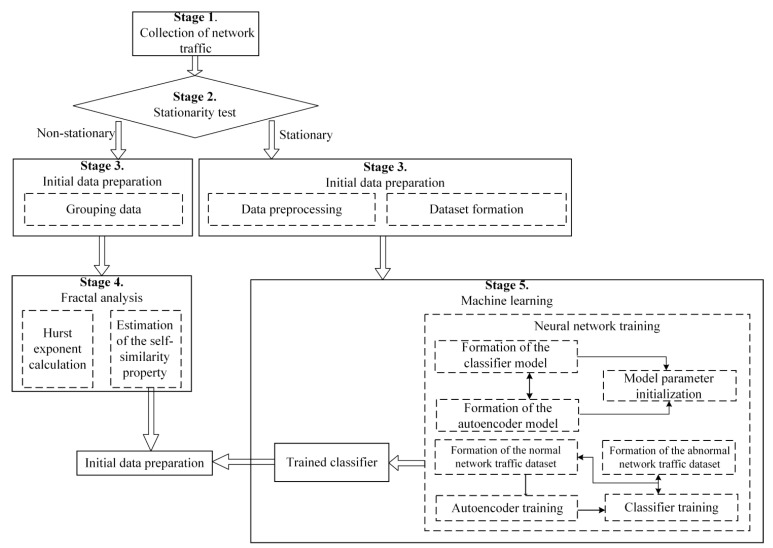
Scheme of the proactive protection system when anomalies are detected.

To detect anomalies in smart power grids from cyber attacks, a proactive protection system is proposed. The scheme of operation of this system contains the following stages (Figure 13):

- collection of network traffic;
- stationarity check;
- preparation of initial data;
- fractal analysis;
- machine learning.

Initially, after traffic is collected, it is checked for stationarity. To calculate the Hurst exponent in stationary traffic, R/S analysis is used, and in non-stationary noisy traffic with time-varying characteristics, DFA analysis is used. The procedure for estimating the Hurst exponent based on R/S and DFA analysis was considered in detail in [6,48].

Next, the detected anomalies are processed in order to predict the fact of the impact of cyber attacks. To do this, a hybrid neural network consisting of an autoencoder and a classifier is used as a machine learning method.

An autoencoder is a feed-forward neural network that reconstructs the input signal at the output. Inside it has a hidden layer, which is the code that specifies the model. The autoencoder is designed to be able to exactly copy the input to the output.

It is proposed to use cells with LSTM as autoencoder layers in the system. The architecture of the LSTM and the algorithm of its operation were considered in detail in [13,49]. Therefore, further, we will consider in more detail the operation of the developed SG protection system against CAs on service data transfer protocols.

### 4.2. Software Implementation

**Figure 14:**
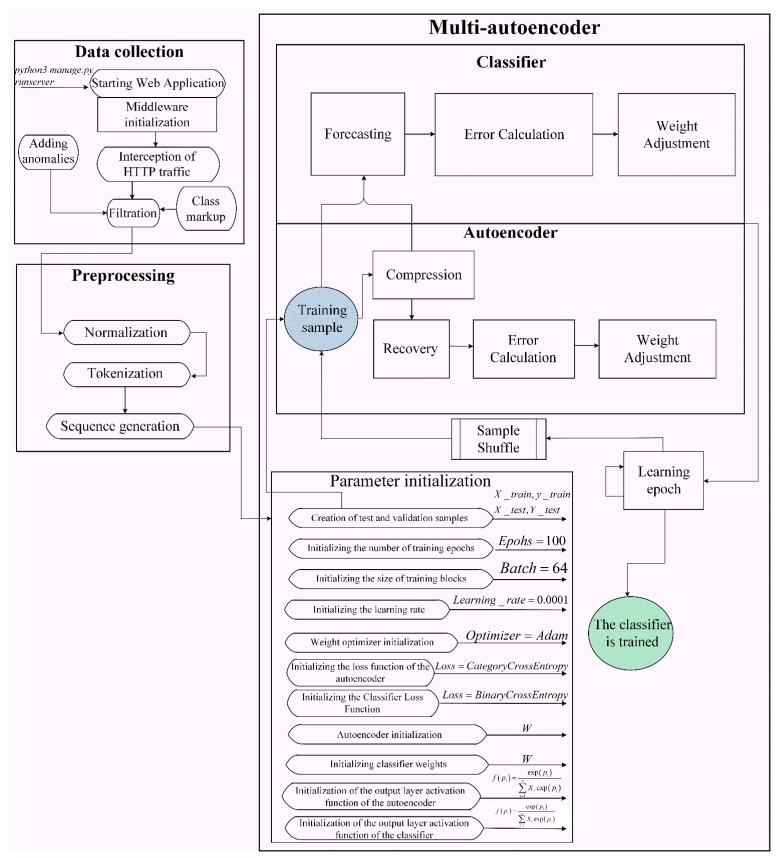
Scheme of functioning of the system for CA detection in stationary traffic.

For the software implementation of the proposed CA detection system (Figure 14), the Python language was chosen. Pandas library, written in the programming languages C, Cython, and Python, was used for data processing and analysis. As a result, Python, despite its high availability, becomes quite a powerful tool for data analysis. It allows one to perform groupings, create pivot tables at a high level, and have easy access to tabular data.

In addition to the Pandas library, we used the NumPy library, which is a lower-level toolkit that allows one to work with multidimensional arrays (tensors) and high-level mathematical functions. The Matplotlib module was used to build graphs. Necessary calculations were carried out in the integrated development environment Jupiter notebook.

In order to intercept the request, a middleware layer (framework Django) was used. It is a middleware framework that allows one to process requests from the browser before they reach the server, as well as to handle responses before they are returned to the browser.

To test the stationarity of traffic, an experiment was carried out, which consisted in plotting the distribution of lengths between two identical characters and estimating the stationarity of the resulting series using the Dickey–Fuller test.

Next, preprocessing and normalization of the resulting sample were performed. Vector representation of characters was used, since the HTTP protocol is a text-based protocol. To implement this method of representation, all the characters available in the dataset were replaced by numeric equivalents (tokens), which have no independent application. Then the words were translated into a sequence of sequences. 

**Figure 15:**
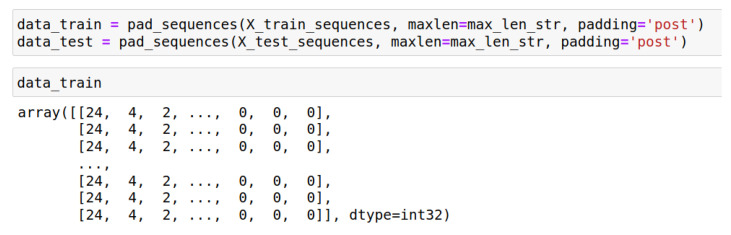
Sequence example.

An example of the resulting array of sequences is shown in Figure 15.

It was taken into account that all sequences must have the same length. If the request length was less than the sequence length, the missing characters were replaced by zeros.

### 4.3. Subsystem for Determining the Stationarity of Network Traffic

Using the Dickey–Fuller test, the value of the autoregression coefficient α is checked in the first-order autoregressive equation *AR*(1):

(7) $$y_{t}=\alpha \cdot y_{t-1}+\varepsilon_{t}\;,$$

where $y_{t}$ is a time series and ε is white noise, t=1,…,T.

1. If $H_{1}:\alpha <1$, then the series $y_{t}$ will be stationary, $y_{t}\sim I\left(0\right)$ and the Ordinary least squares (OLS) estimator $\hat{\alpha }$ will have a normal distribution with zero mean and variance $\hat{\alpha }$.

To test the unit root hypothesis, an OLS estimator $\hat{\alpha }$ is constructed:

(8) $$\hat{\alpha }=\frac{\sum_{t=1}^{T}y_{t-1}y_{t}}{\sum_{t=1}^{T}y_{t-1}^{2}}$$

and the corresponding *t*-statistic:

(9) $$t_{\alpha }=\frac{\hat{\alpha }-1}{S/\sqrt{\sum_{t=1}^{T}y_{t-1}^{2}}}$$

where $S^{2}=T^{-1}\overset{T}{\underset{t=1}{\sum }}{\left(y_{t}-\hat{\alpha }y_{t-1}\right)}^{2}$ is the estimated variance of the residuals.

If the value of statistic $t_{\alpha }$ lies to the left of the critical value at the 5% significance level, i.e., $t_{\alpha }<t_{\mathrm{critical}}^{5\%}$, then the time series is stationary.

2. If $H_{0}:\alpha =1$, then the distribution of this estimate will no longer be normal, and the process $y_{t}$ will be non-stationary with a time-dependent variance $y_{t}\sim I\left(1\right)$. In this case, to model the dynamics of such a series, it is necessary to use its first difference $\Delta y_{t}=y_{y}-y_{t-1}$. Under the null hypothesis, the normalized bias statistic $T\left(\hat{\alpha }-1\right)$ and the *t*-statistic $t_{\alpha }$ have non-standard marginal Dickey–Fuller distributions:

(10) $$T\left(\hat{\alpha }-1\right)\Rightarrow \frac{\int_{0}^{1}W\left(r\right)dW\left(r\right)}{\int_{0}^{1}W^{2}\left(r\right)dr}\;\mathrm{and}\;t_{\alpha }\Rightarrow \frac{\int_{0}^{1}W\left(r\right)dW\left(r\right)}{\sqrt{\int_{0}^{1}W^{2}\left(r\right)dr}}$$

where W(r) is the standard Wiener process (Brownian motion).

If $t_{\alpha }>t_{\mathrm{critical}}^{5\%}$, then the time series is non-stationary.

### 4.4. Subsystem of Anomaly Analysis in a Stationary and Non-Stationary Network

**Figure 16:**
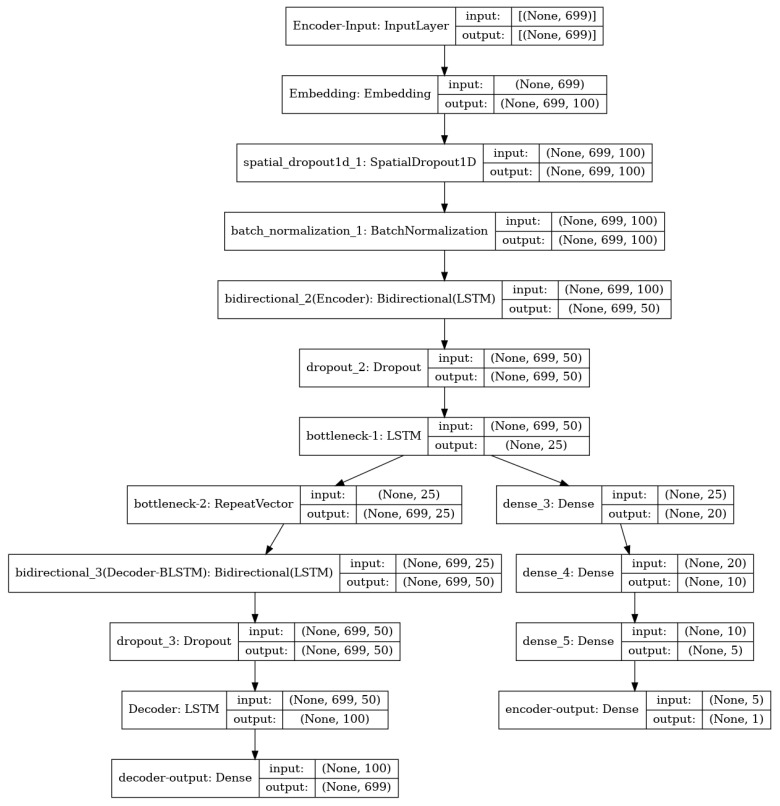
Hybrid neural network model.

To detect anomalies in a stationary network, it is proposed to use a hybrid neural network model (Figure 16) created on the TensorFlow framework using the Python language.

The autoencoder model consists of Gated Recurrent Units (GRUs), which are elements of the LSTM neural network. Data up to 699 symbols are fed to the input of the neural network.

The neural network has several output layers. The output layer of an autoencoder has exactly the same dimension as the input layer. The classifier has one output layer. It determines if the request is anomalous or legitimate.

## 5. Experimental Evaluation of the System

**Figure 17:**
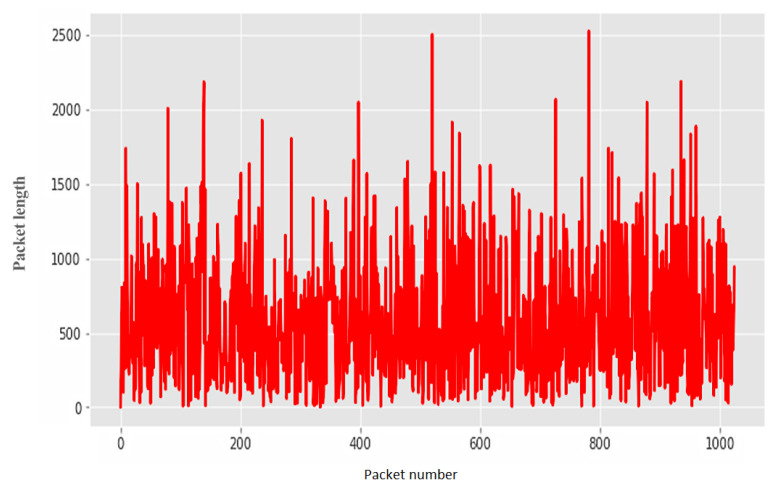
Legitimate traffic.

**Figure 18:**
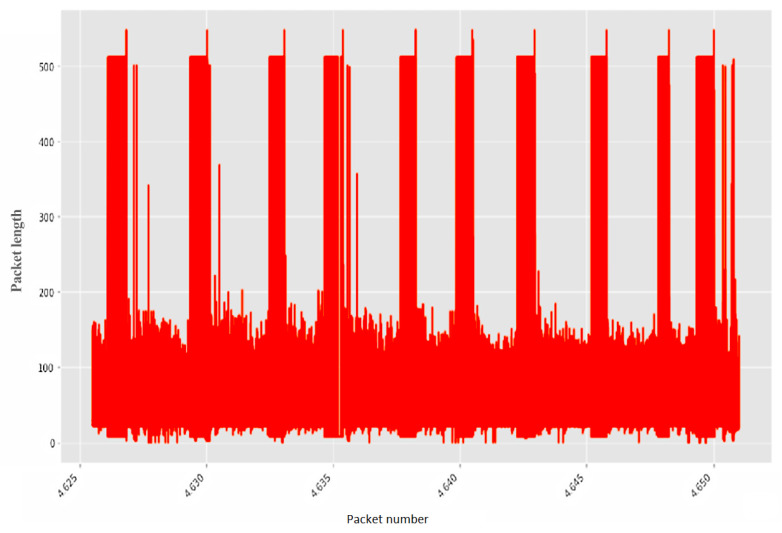
Abnormal traffic.

Non-stationary network traffic received using the created cyber polygon was divided into legitimate (Figure 17) and anomalous (Figure 18) samples.

The analysis showed that in order to detect anomalous behavior in traffic, it is enough to analyze its main parameters. There is no need to study the contents of each packet. Examples of anomalies detected based on traffic telemetry analysis are a sudden increase in traffic from a workstation or a change in its structure compared to normal daily rates for a given network device.

For each sample, the Hurst exponent was calculated using the R/S algorithm.

**Figure 19:**
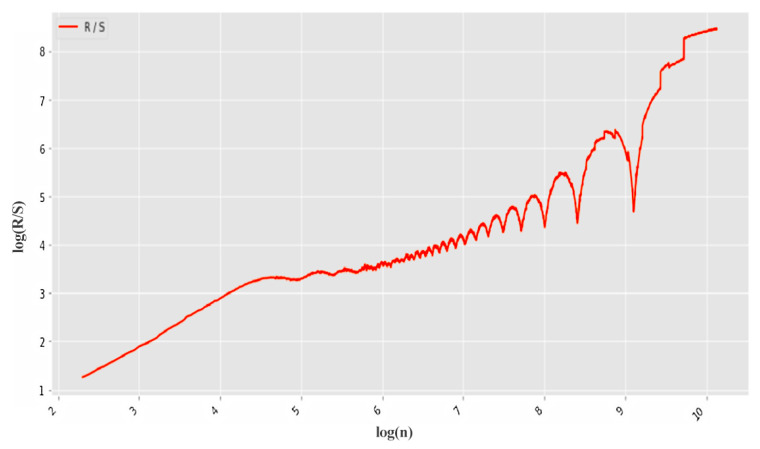
R/S versus time on a logarithmic scale.

Figure 19 depicts an example of calculating *H* for non-stationary traffic, which showed the result *H* = 1.378. 

In turn, the Hurst exponent exceeding the maximum value of 1 confirms the presence of anomalies in network traffic.

**Figure 20:**
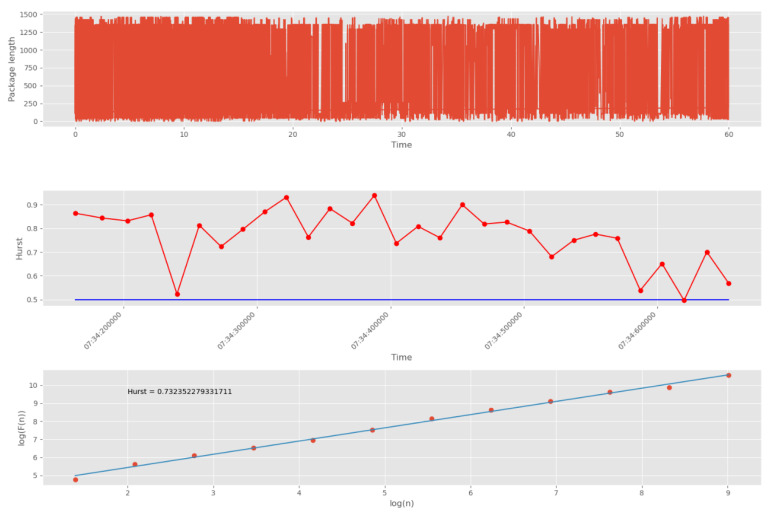
Computing *H* for legitimate UDP traffic.

To quickly find anomalies caused by CAs, the network stream is first divided into groups. The Hurst exponent is then calculated for each of the groups. The result of such processing is shown in Figure 20. In this example, 10,000 points were divided into 20 groups.

The threshold corresponding to the white noise boundary (*H* = 0.5) is indicated by the blue line. The points on the second graph correspond to the number of packet groups (30 points in total). On the third graph, the dots correspond to the number of scales (12 dots in total). The number of scales affects the accuracy and duration of the algorithm. Increasing the number of scales increases accuracy, and decreasing the number of scales decreases accuracy.

Figure 20 shows the Hurst exponent for all groups of packages, which is above the 0.5 mark. This indicates self-similarity properties for each of the network traffic groups. The third graph (logarithmic regression graph) shows the Hurst exponent for all data, which confirms the presence of fractal properties and repetitive processes.

**Figure 21:**
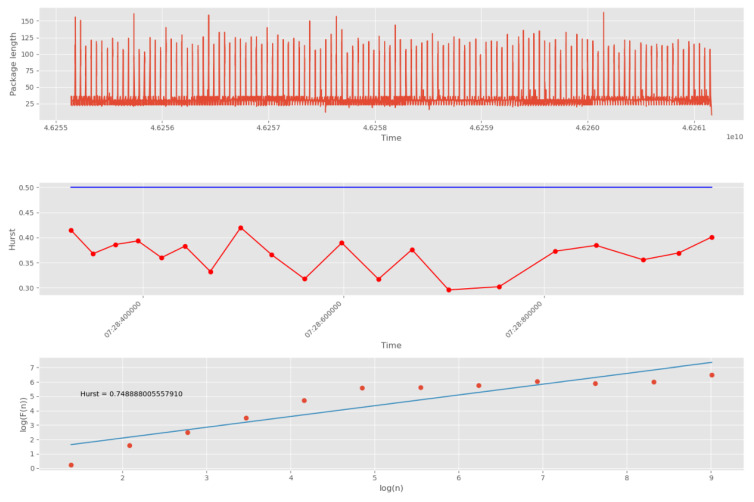
Computing *H* for abnormal UDP traffic.

Next, we tested abnormal network traffic received during a DDoS attack and a cyberattack “Scanning the network and its vulnerabilities”. The result of calculating *H* for this anomalous traffic is shown in Figure 21. It can be seen that in this case the self-similarity property is violated, since the Hurst exponent at each of the intervals has a value less than the threshold of 0.5.

**Figure 22:**
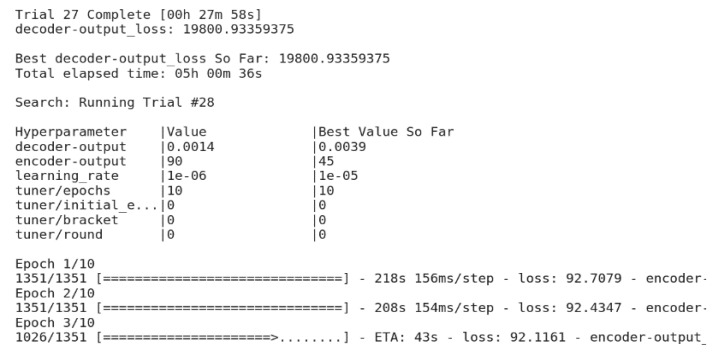
Selection of neural network hyperparameters.

The training dataset includes both legitimate and anomalous traffic. Only legitimate traffic was fed to the input of the autoencoder. The classifier input received legitimate and anomalous traffic, as well as hidden latent representations received from the autoencoder after encoding the information. The results of the selection of the neural network parameters are shown in Figure 22. The selection of parameters was carried out in such a way that the loss function during training of the autoencoder decreased, while the accuracy of the classifier grew.

**Figure 23:**
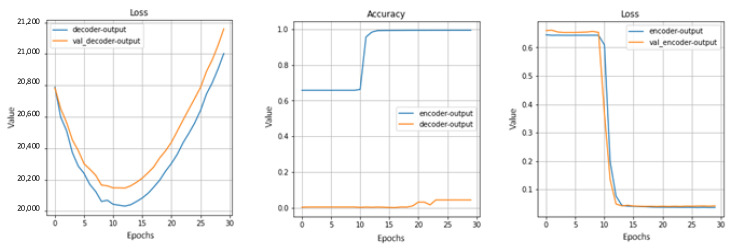
Decoder and classifier training on 30 epochs.

Figure 23 demonstrates the results of estimating accuracy growth and loss reduction over 30 training epochs.

**Figure 24:**
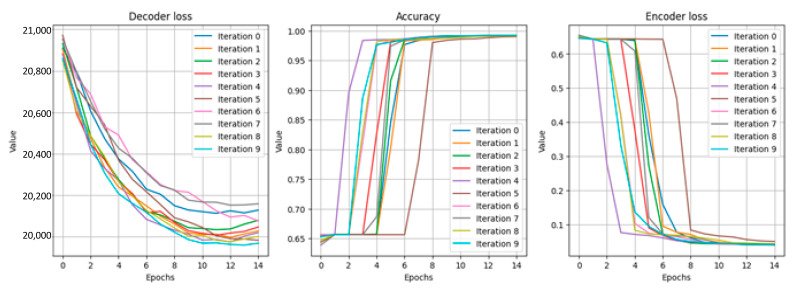
10-fold cross-validation at 15 epochs.

To empirically evaluate the generalizing ability of the neural network, a 10-fold stratified K-Folds cross-validator was used on unique data with the most uniform use of available data (Figure 24).

After training the neural network, an experiment was conducted to assess the accuracy and completeness of the detection of known anomalies. First, a dataset with CAs of the same type was used as in the dataset when training the model. The anomaly detection result showed a value of 96.9%.

Then a new dataset was formed containing CAs previously unknown to the classifier (“0-day” attacks). The algorithm recognized 80% of previously unknown attacks. At the same time, it determined that 99% of legitimate requests are not anomalous.

**Figure 25:**
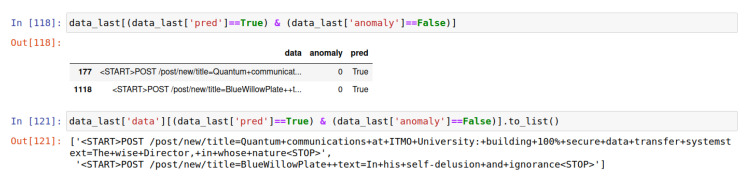
Examples of false positives in the system.

It has been observed that the system allows false positives. In particular, two requests were dropped by the neural network. Examples of such false positives in the system are shown in Figure 25.

Given the fact that the dataset contained 57,000 queries, of which 20,000 were anomalous, the value of 2 is not a significant drawback of the proposed approach.

## 6. Discussion

Experiments have shown that SG network traffic has fractal properties. In other words, in large volumes this traffic has the property of self-similarity.

In addition, experiments have shown that the proposed proactive SG protection system upon detection of CAs based on the assessment of self-similarity of system functioning parameters using fractal indicators and predicting the fact of the impact of CAs by applying the proposed structure of the LSTM neural network has fairly high efficiency in detecting both known and unknown CAs. The probability of detecting known CAs is 0.96, and “0-day” attacks is 0.8.

A comparative evaluation of the proposed approach was carried out with intrusion detection systems (IDS) and intrusion prevention systems (IPS), which were based on signature [50], statistical [51], and machine learning methods [52,53].

The results of this assessment are shown in Table 4. It demonstrates the detection rate (in seconds) and detection accuracy of known and unknown CAs types. In addition, the table indicates what type of traffic the method is suitable for.

Table 4 shows that signature methods and the proposed method are the fastest in terms of detection rate. Also, because signature methods use predefined rules, they have the highest accuracy in detecting known attacks. However, their accuracy in detecting unknown attacks is very low. A value of 0.5 indicates that this accuracy corresponds to the law of equiprobability.

Statistical methods lose out to signature methods in terms of detection rate and accuracy, since they use accumulated statistics. However, sometimes they are able to detect unknown attacks.

Machine learning methods are quite diverse and well-developed. Their effectiveness depends on the classification and clustering models they use. In the works [52,53] considered in Table 4, the SVM, Gaussian Naive Bayes, and Decision Tree models were used. In these methods, it is necessary to train models on control samples. Therefore, machine learning methods lose signature methods in detection rate. However, they have higher accuracy in detecting unknown attacks.

The proposed method has a detection rate, similar to the signature methods, and the accuracy corresponds to the values, similar to the machine learning methods. At the same time, it retains its effectiveness when working with non-stationary traffic, which is most typical for SG traffic. The remaining methods work well only in the case of stationary traffic.

It should be noted that, at present, for the continuous controlling of the transfer of technological and other information in SG, the systems built on distributed ledger and blockchain technologies, based on smart contracts, are actively used [54,55]. The use of such solutions makes it possible to protect the information transmitted in SG from CAs aimed at violating its confidentiality and integrity. However, these technologies do not provide early detection of CAs, their classification, and protection of SG network devices from CAs, the implementation of which is aimed at learning the SG structure and the subsequent violation of the performance of the network and its elements.

The proposed approach to proactive protection of SG from CAs can be implemented in many existing IDS and IPS, whose main task is to analyze internal data streams, searching in them for bit sequences that may represent malicious actions or events, as well as monitoring system logs. It increases the probability of detecting unknown CAs by using the autoencoder and LSTM networks, reducing the probability of false positives and the time and the amount of RAM involved in analyzing the network traffic. Thus, the disadvantages of existing IDS and IPS, based on rules, as well as signature and anomaly technologies are leveled.

It should be noted that the conducted studies only demonstrate the effectiveness of the proposed proactive system for predicting and detecting CAs in the SG network. It can be considered as an attack detection system that combines the advantages inherent in signature, statistical, and machine learning methods, and is devoid of their inherent disadvantages. At the same time, it expands the scope of attack detection methods by extending them to a non-stationary type of traffic.

## 7. Conclusions

The article discusses a new approach to the operation of a system for protecting smart power grids from CAs on service data transfer protocols, based on the detection of anomalies in network traffic by evaluating its self-similarity property, detecting cyber attacks in anomalies in real or near real-time, their classification and acceptance effective protection measures using LSTM and GRU cells. Fractal analysis, mathematical statistics, and neural networks with long short-term memory were used as tools in the development of this protection system.

The proposed system is based on the application of the main provisions of the theory of fractals and the use of self-similarity assessment methods proposed by this theory, such as the Dickey–Fuller test, R/S analysis, and the DFA method. When testing fractal methods that make it possible to study long-term dependencies in network traffic, the DFA method is more efficient than R/S analysis due to its ability to process not only stationary, but also non-stationary series with high accuracy. Its joint application with LSTM networks can significantly increase the probability of detecting CAs.

The experimental evaluation of the proposed approach showed that, compared with many other approaches, one of the main advantages of fractal analysis is its speed, as well as the ability to detect anomalies in traffic of any kind. Only an increase in the number of processed data transfer protocol header parameters (packet length, flags, and others) leads to an increase in the calculation time. At the same time, the proposed system demonstrated a fairly high probability of detecting CAs, reaching a value of 0.96 for known attacks and 0.8 for previously unknown attacks.

The specificity of the proposed system is that the detection of CAs is performed using an autoencoder trained on the basis of the reference data of the SG operation and the information exchange in it, taking into account all deviations from the regular operation of the SG. During operation, the autoencoder is additionally trained by a reasonable neural network, i.e., the result is a generative adversarial network in which neural networks learn from each other.

Further studies are associated with the integration of the proposed system with other known protection systems, as well as with the attack detection methods available in the arsenal of computer security systems.

## Figures and Tables

**Table 1:** The main attributes included in the dataset.

#	Attribute Name	Comments
1	Bwd.Packet. Length.Max	The maximum packet length (in bytes) in the backward direction
2	Bwd.Packet. Length.Mean	The mean packet length (in bytes) in the backward direction
3	Bwd.Packet. Length.Min	The minimum packet length (in bytes) in the backward direction
4	Bwd.Packet. Length.SD	The standard packet length deviation (in bytes) in the backward direction
5	Destination.IP	The destination IP address
6	Destination.Port	The destination port number
7	Flow.Duration	The total flow duration
8	Flow.ID	A flow identifier. It has the following format: Source.IP-Destination.IP-Source.Port-Destination.Port-Protocol
9	Fwd.Packet. Length.Max	The maximum packet length (in bytes) in the forward direction
10	Fwd.Packet. Length.Mean	The mean packet length (in bytes) in the forward direction
11	Fwd.Packet. Length.Min	The minimum packet length (in bytes) in the forward direction
12	Fwd.Packet. Length.SD	The standard packet length deviation (in bytes) in the forward direction
13	Packet.Length.Mean	The mean length value of the packets registered in the flow (both forward and backward directions)
14	Protocol	The transport layer protocol number identification (value is 6 for the TCP protocol and 17 for the UDP protocol)
15	Source.IP	The source IP address of the flow
16	Source.Port	The source port number
17	Timestamp	Packet capture moment. The value is stored in the following format: Dd/mm/yyyy HH:MM:SS
18	Total.Backward.Packets	The total number of the backward packets
19	Total.Fwd.Packets	The total number of the forward packets
20	Total.Length.of. Backward	The total number of bytes in the backward direction obtained from all the flow (all the packets have been transmitted)
21	Total.Length.of. Fwd	The total number of bytes in the forward direction received from all the flow (all packets have been transmitted)

**Table 2:** Additional dataset attributes.

#	Attribute Name	Comments
1	ACK.Flag.Count	The number of times the ACK (Acknowledged) flag for packets sent in both directions was 1
2	FIN.Flag.Count	The number of times the FIN flag for sent packets was 1. Normally the operation ends with the transmission of a packet in which the FIN is 1
3	RST.Flag.Count	The number of times the RST (Reset) flag for packets sent in both directions was 1
4	SIN.Flag.Count	The number of times the SIN (Synchronization) flag for packets sent in both directions was 1

**Table 3:** Network protocol parameters.

No.	Name	Type	Description
1	dbytes	integer	Destination to source transaction bytes
2	dintpkt	float	Destination interpacket arrival time
3	djit	float	Destination jitter (mSec)
4	dload	float	Destination bits per second
5	dloss	integer	Destination packets retransmitted or dropped
6	dmeansz	integer	Mean of the packet size sent by destinations
7	dpkts	integer	Destination to source packet count
8	dsport	integer	Destination port number
9	dstip	nominal	Destination IP address
10	dtcpb	integer	Destination TCP base sequence number
11	dttl	integer	Destination to source time to live value
12	dur	float	Record total duration
13	dwin	integer	Destination TCP window advertisement value
14	ltime	timestamp	Record last time
15	proto	nominal	Transaction protocol
16	res_bdy_len	integer	Actual uncompressed content size of data
17	sbytes	integer	Source to destination transaction bytes
18	service	nominal	http, ftp, smtp, ssh, dns, ftp-data, irc and others
19	sintpkt	float	Source interpacket arrival time
20	sjit	float	Source jitter (mSec)
21	sload	float	Source bits per second
22	sloss	integer	Source packets retransmitted or dropped
23	smeansz	integer	Mean of the packet size sent by sources
24	spkts	integer	Source to destination packet count
25	sport	integer	Source port number
26	srcip	nominal	Source IP address
27	state	nominal	Indicates to the state and its dependent protocol
28	stcpb	integer	Source TCP base sequence number
29	stime	timestamp	Record start time
30	sttl	integer	Source to destination time to live value
31	swin	integer	Source TCP window advertisement value
32	synack	float	TCP connection setup time
33	tcprtt	float	TCP connection setup round-trip time
34	trans_depth	integer	Represents the pipeline depth into connection

**Table 4:** Comparative analysis of methods for detecting cyber attacks.

Method Name	Detection Rate (s)	Detection Accuracy		Traffic Type	
		Known Attacks	Unknown Attacks	Stationary	Non-Stationary
Signature methods [50]	5	0.99	0.5	+	-
Statistical methods [51]	30	0.92	0.6	+	-
Machine learning methods [52,53]	28	0.72–0.97	0.8	+	-
**Proposed method**	**5**	**0.96**	**0.8**	**+**	**+**

## Data Availability

Not applicable.
